# Amoebic ulcer of the male genitala: A rare case report

**DOI:** 10.4103/2589-0557.75009

**Published:** 2010

**Authors:** Indrani Mohanty, Prasenjeet Mohanty, Satyadarshi Patnaik, Pritilata Panda

**Affiliations:** Department of Microbiology, The Maharaja Krishna Chandra Gajapati Medical College, Berhampur, Orissa, India; 1 Department of Skin & V.D, The Maharaja Krishna Chandra Gajapati, Medical College Berhampur, Orissa, India

**Keywords:** Amoebic ulcer, Entamoeba histolytica, glans penis, homosexual

## Abstract

Amoebic ulcer of the penis is a very rare clinical entity. We report a case of amoebic ulcer of the glans penis in a 47-year-old male homosexual, symptomatic with severe pain and foul-smelling hemopurulent discharge of acute onset. He had received systemic antibiotics like ciprofloxacin and azithromycin prior to presentation with no improvement. Diagnosis was confirmed by wet mount microscopic examination of the discharge. The patient responded well to a course of metronidazole.

## INTRODUCTION

Amoebiasis is still a great public health problem, especially in several underdeveloped regions of the world. It is the most widespread parasitic disease throughout the world and is endemic in several African, Asian, and Latin American countries.[1] Genital amoebiasis, a rare extra-intestinal manifestation of this disease is often missed by the clinicians. Genital amoebiasis should be considered in the differential diagnosis of benign and malignant ulcers of the penis.[2]

## CASE REPORT

**Figure 1:**
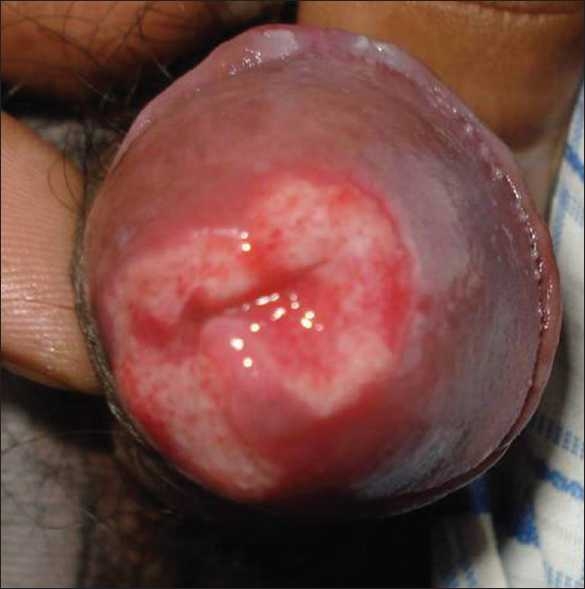
Photograph of patient showing amoebic ulcer of glans penis prior to any treatment

A 47-year-old male, presented with an extremely painful ulcer of 15 days duration over the glans penis, surrounding the urethral orifice. The ulcer had a well-demarcated border with a raised erythematous rim [Figure 1]. The entire penis was edematous and associated with foul-smelling hemopurulent exudates. Bilateral inguinal lymphadenopathy was present. The lesion started as multiple superficial ulcers, which coalesced to form a spreading ulcer. There was history of homosexuality. The patient had already been treated with ciprofloxacin and azithromycin by local practitioners without any response.

**Figure 2:**
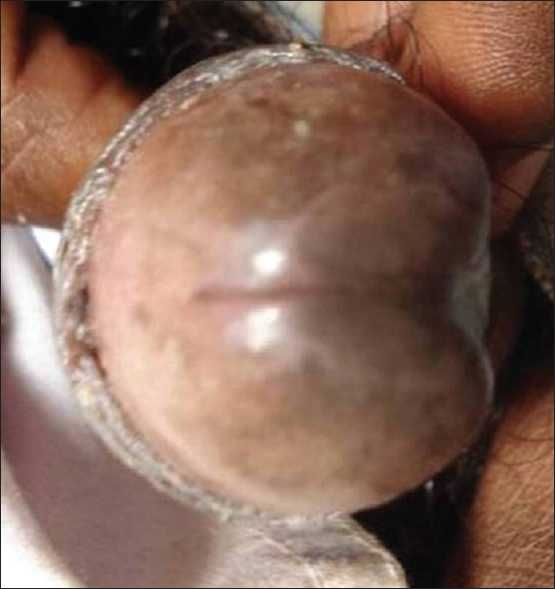
Photograph of patient showing a healed lesion on the glans penis, 7 days after treatment with metronidazole

Complete hemogram, blood sugar, urine analysis, and stool examination were normal. Venereal Disease Research Laboratory (VDRL) and HIV antibody tests were non-reactive. Scrape cytology was performed, which showed the presence of inflammatory cells, and no atypia or dysplastic cells were seen. A wet mount of the hemopurulent discharge revealed the presence of trophozoites of Entamoeba histolytica. The parasite was not seen on stool examination. A two-week course of metronidazole was prescribed with dramatic regression of the ulcer in 1 week and complete regression in 2 weeks [Figure 2].

## DISCUSSION

Amoebic infection of the genitalia in both sexes is an extremely rare and unusual ectopic site.[3] In males the classic presentation is of a painful penile ulcer, which progresses rapidly with a mucopurulent discharge. The best differentiating feature between penile cancer and amoebic ulcer appears to be the absence of pain in patients with cancer.[4] Amoebic ulcers are serpiginous, with distinct raised, thickened, often undermined edges and erythematous rim, hemopurulent exudates, and necrotic slough. Pain is intense and regional adenitis is usual.[5] The mode of infection is by direct inoculation during vaginal or anal intercourse with a person suffering from amoebic dysentery.[6] Our patient was a homosexual and may have acquired the infection from anal intercourse with a person suffering from intestinal amoebiasis.

Entamoeba histolytica has the capacity of destroying all tissues of the human body. It does this by means of several virulence factors: adhesion molecules, toxins, contact-dependent cytolysis, protease, and phagocytic activity. Damage is produced by the trophozoites that penetrate the mucosa and adhere to the host cells.[4]

Diagnosis can be made on the typical appearance of the ulcer, its acute onset, rapid progression, and associated intense pain. Wet mount microscopy of ulcer exudates demonstrates trophozoites. There was no associated intestinal or cutaneous amoebiasis in our case. Treatment of this unusual disease is with metronidazole and antibacterial agents to prevent secondary infection. In case of massive destruction of the penis, surgical intervention may be necessary.[2]
